# Withdrawn: CIP2A promotes the survival of renal clear cell carcinoma Caki‐2 cells

**DOI:** 10.1002/2211-5463.12870

**Published:** 2021-02-03

## Abstract

The above article, published online on April 27, 2020, in Wiley Online Library (wileyonlinelibrary.com) as an Accepted Article (https://doi.org/10.1002/2211‐5463.12870), has been withdrawn by agreement between the authors, the journal Editor in Chief Miguel De La Rosa, and John Wiley and Sons Ltd. The withdrawal has been agreed because the authors are not responding to requests to finalize their article for publication in the journal as the Version of Record.

